# Antibiotics and Other Drugs Removal by the CytoSorb^®^ Haemoadsorber: A Systematic Review of Available Evidence

**DOI:** 10.3390/antibiotics15040409

**Published:** 2026-04-17

**Authors:** Sara Kenda, Jakob Gubenšek, Tomaž Vovk

**Affiliations:** 1 Faculty of Pharmacy, University of Ljubljana, Askerceva 7, 1000 Ljubljana, Slovenia; sara.kenda@sbng.si; 2 General Hospital Dr. Franc Derganc Nova Gorica, Padlih Borcev 13a, 5290 Sempeter Pri Gorici, Slovenia; 3 Department of Nephrology, University Medical Center Ljubljana, Zaloska 7, 1000 Ljubljana, Slovenia; jakob.gubensek@kclj.si; 4 Faculty of Medicine, University of Ljubljana, Vrazov trg 2, 1000 Ljubljana, Slovenia

**Keywords:** clearance, CytoSorb, drugs, drug binding, haemoadsorbent, hemoadsorption, sepsis, percent removal, pharmacokinetics

## Abstract

**Background/Objectives**: Haemoadsorption has recently emerged as an extracorporeal treatment option for sepsis, septic shock, intoxications, and cardiac surgery to modulate dysregulated inflammatory responses or remove a wide range of circulating molecules. To ensure appropriate clinical use of the CytoSorb^®^ haemoadsorber, it is essential to understand the extent to which specific drugs are adsorbed by the device. **Methods**: We conducted a systematic literature review using the PubMed and Ovid MEDLINE database to identify studies on drug binding to the CytoSorb^®^ haemoadsorber, including both in vivo and in vitro studies. Publications in English language, available up to 31 December 2025 that reported or enabled calculation of percentage of drug removal, CytoSorb^®^ clearance or half-life during CytoSorb^®^ therapy were included. Records were screened, eligibility and quality were assessed, and data were extracted independently by two reviewers. **Results**: We found that 26 studies reported on the binding of 56 drugs to CytoSorb^®^, with most available information relating to antibiotics used in the treatment of sepsis and septic shock. CytoSorb^®^ appears to remove vancomycin and linezolid but not meropenem, although data for other antibiotics are insufficient to assess clinical relevance. Data on the removal of anticoagulant and antithrombotic drugs with CytoSorb^®^ before and during cardiac surgery indicate that using this procedure to reduce complications associated with apixaban and ticagrelor is feasible and safe. The available evidence on the use of CytoSorb^®^ for drug poisoning is of very low quality. **Conclusions**: Although the number of studies on drug binding to the CytoSorb^®^ is increasing, the review is limited by the marked heterogeneity among the included studies. It is advised to use therapeutic drug monitoring whenever possible during CytoSorb^®^ treatment. Research of binding of drugs to CytoSorb^®^ is crucial for its safe and effective clinical use, but adequate methodology is necessary.

## 1. Introduction

Haemoadsorption with the CytoSorb^®^ device (CytoSorbents Corporation, Monmouth Junction, NJ, USA) is a rapidly evolving method of extracorporeal blood purification designed to modulate dysregulated inflammatory responses and remove a wide range of circulating molecules by adsorption onto biocompatible polymer beads. CytoSorb^®^ cartridges contain porous polystyrene–divinylbenzene beads coated with polyvinylpyrrolidone, which adsorb substances mainly in the mid-molecular weight range (5–60 kDa) through hydrophobic and ionic interactions, hydrogen bonding, and van der Waals forces. This mechanism enables the removal of pro-inflammatory cytokines, metabolic waste products, and potentially various therapeutic drugs when used in extracorporeal circuits such as continuous renal replacement therapy (CRRT), extracorporeal membrane oxygenation (ECMO), or cardiopulmonary bypass (CPB) systems [1].

Originally introduced for severe systemic inflammation in sepsis, CytoSorb^®^ has been investigated in various clinical contexts due to its broad adsorption capacity and favourable safety profile. Its applications include: (i) adjunctive therapy in sepsis and septic shock, aiming to mitigate cytokine storm and haemodynamic instability [2,3]; (ii) management of severe drug intoxications and toxin removal [4]; and (iii) perioperative drug clearance before or during complex cardiovascular surgery, particularly in patients on antithrombotic medications [5,6,7].

In sepsis and septic shock, conditions characterised by an overwhelming release of cytokines and other inflammatory mediators, several observational studies and meta-analyses have documented reductions in pro-inflammatory biomarkers (e.g., IL-6, TNF-α) and improvements in haemodynamic parameters with CytoSorb^®^ adjunctive therapy [8,9,10]. However, evidence regarding mortality benefit remains negative [2,11]. Although CytoSorb^®^ use can have some therapeutic advantage, it may also imply a significant risk for the patient when unwanted removal of potentially life-saving medications occurs [12].

Beyond septic states, extracorporeal haemoadsorption has been used in acute intoxications, including cases of 3,4-methylenedioxymethamphetamine (MDMA) overdose [13], venlafaxine-induced cardiovascular collapse [14], and complex multi-drug toxicities, where adsorptive removal of the offending agents is associated with rapid decreases in measured serum concentrations and clinical stabilisation. Although much of the evidence comes from case reports, they highlight the potential benefits of haemoadsorption in toxicological emergencies that are resistant to standard detoxification methods [4].

Finally, in the context of cardiovascular surgery, intraoperative or perioperative integration of CytoSorb^®^ into the CPB circuit has been investigated as a strategy to attenuate inflammatory activation associated with CPB and to remove anticoagulants or antiplatelet agents such as ticagrelor and rivaroxaban before emergency surgery. Clinical data suggest that CytoSorb^®^ is feasible and safe, but has not demonstrated consistent attenuation of inflammatory activation or improvement in clinical outcomes in unselected cardiac surgery populations [15,16]. In the context of anticoagulant and antiplatelet therapy removal, CytoSorb^®^ demonstrated efficacy in adsorbing ticagrelor and rivaroxaban during emergency cardiac surgery, resulting in reduced bleeding complications, lower transfusion requirements and decreased re-thoracotomy rates in patients at a high risk of bleeding [6,17].

Given the widespread use of CytoSorb^®^, comprehensive knowledge of the literature on drug haemoadsorption is essential. In our study, we conducted a systematic review of published data on drug binding to the CytoSorb^®^ haemoadsorber. We evaluated the identified articles according to their level of evidence and categorised them based on the various clinical applications of CytoSorb^®^. Based on the research data, we have prepared recommendations for further studies to ensure the most conclusive demonstration of drug binding to CytoSorb^®^. For active substances with sufficiently robust data, we also provide clinical considerations on their administration during CytoSorb^®^ therapy. To our knowledge, this is the first systematic review to include studies on drug binding to CytoSorb^®^ in vitro and in vivo, from which we were able to obtain percentage removal, clearance, and half-life.

## 2. Results

### 2.1. Study Selection

Through the initial database search, a total of 1651 studies were identified. All were screened by title and abstract, resulting in the exclusion of 1031 studies. Full-text evaluation was done for 69 studies, in addition to the 11 studies identified through the cited references. We divided all studies into four categories based on CytoSorb^®^ application: (i) treatment of sepsis and septic shock, (ii) treatment of intoxications, (iii) removal of drugs before cardiac surgery, and (iv) other indications.

### 2.2. Study Characteristics

Out of the 80 studies, 21 studies described treatment of sepsis and septic shock, 22 focused on treatment of intoxications, 27 examined drug removal before cardiac surgery, 2 addressed immunosuppressive drugs, 2 were in vitro studies covering various topics, and 6 reported on different indications but did not meet the inclusion criteria. Additionally, we reviewed the selected studies to determine whether they contained relevant data from which we could calculate percentage removal, the difference in clearance between treatment with and without CytoSorb^®^, or the half-life in the case of treatment with or without CytoSorb^®^. Collectively, we included 27 studies in our systematic review: 10 in the sepsis and septic shock group, 8 in the intoxications group; 7 in the cardiac surgery group; and 4 in the other group. Two articles covered more than one area. The quality of the selected study was assessed using different risk of bias tools depending on the study type. The results are presented in Supplementary Materials (Tables S3–S6).

**Figure 1:**
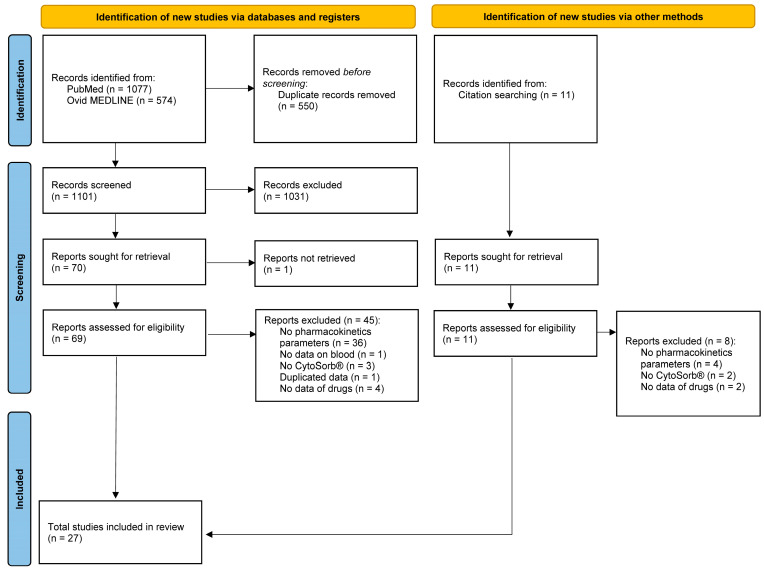
Flowchart of study identification and inclusion process.

The results are presented by field of application in tables that describe the data provided above, including the types of studies conducted and the assessed level of evidence. Flowchart of study identification and inclusion is presented in Figure 1.

### 2.3. Sepsis and Septic Shock

The treatment of sepsis and septic shock may be challenging due to the possible adsorption of antibiotics and antifungal drugs during CytoSorb^®^ therapy, which could reduce therapeutic effectiveness of these antimicrobial agents. Table 1 and Table 2 list studies that investigate drug binding to CytoSorb^®^ in the context of sepsis and septic shock treatment. For in vitro studies, the maximum percentage removal is reported, whereas for in vivo studies, the range of percentage removal is provided.

Several studies describe the binding of active substances to CytoSorb^®^, which is used in the treatment of sepsis and septic shock, but no randomised clinical trials on antibiotics dosing are available. Most data come from in vitro studies and studies on a porcine model, which, as explained in the Discussion, cannot be directly extrapolated to in vivo use in humans. In the field of glycopeptide antibiotics, we have data for vancomycin and teicoplanin. A study by Scharf et al. demonstrates that vancomycin needs to be replaced, specifically 500 mg in the first two hours. This study involved seven patients receiving vancomycin as a continuous infusion, with 20 CytoSorb^®^ treatments performed [19]. Additionally, a case report on vancomycin describes a slightly different scenario, indicating that replacement is necessary only with bolus infusion, but not with continuous infusion [18]. For teicoplanin, a case report suggests that replacement is not necessary [18], and a study in pigs found that total clearance of teicoplanin compared to endogenous clearance is increased by 30.7% when using CytoSorb^®^ [12]. Based on the available data on glycopeptides, vancomycin should be replaced, while the data on teicoplanin binding are insufficient to provide recommendations about dosing. In the area of beta-lactam antibiotics, the most data are available for meropenem. All studies conducted show that meropenem does not significantly bind to CytoSorb^®^, so replacement is not required. One of these studies was conducted in paediatric patients, data are also available on the difference in clearance between CytoSorb^®^ and CRRT [21]. Piperacillin binding data likewise indicate that piperacillin does not bind to CytoSorb^®^ [12,22]. A case report investigating the beta-lactam antibiotic cefiderocol reports drug binding to Cytosorb^®^. The study shows that CytoSorb^®^ clearance changes over time, but at certain points it exceeds the clearance of the haemofilter [20]. For the other beta-lactam antibiotics, the available data are currently insufficient to demonstrate that removal by CytoSorb^®^ is clinically significant [12,21]. Data on the binding of beta-lactam antibiotics to CytoSorb^®^ are sufficient to make recommendations only for meropenem, while further in vivo research is required for the other antibiotics. For aminoglycosides, CytoSorb^®^ has been shown to have no significant effect on their removal [12,21,34], but there is no enough data to provide recommendations about drug dosing. Regarding other antibiotics, recommendations can be made for linezolid, which is significantly removed. In a case report, plasma concentrations below the therapeutic range according to TDM are reported [23]. Additionally, the study on pigs demonstrates that linezolid clearance increases by 114.6% with CytoSorb^®^ [12]. Most of the results for other active substances used in sepsis and septic shock come from studies on pigs, but no recommendations for dosing in humans can be seen in this research [12]. Among antifungals, CytoSorb^®^ has been reported to remove fluconazole, posaconazole, and liposomal amphotericin B [12]. Another study found that isavuconazole concentrations, according to TDM, were not different when CytoSorb^®^ was used [40]. Data for antivirals are very limited. An in vitro study with remdesivir showed 100% removal during the first 60 min, suggesting that remdesivir and CytoSorb^®^ should not be used concomitantly [36].

### 2.4. Intoxications

There are limited data on the use of CytoSorb^®^ in the treatment of various poisonings. All available data consist of case reports and in vitro studies. Currently, there are no studies comparing the clearance achieved by CytoSorb^®^ with endogenous clearance or dialysis clearance, but there are some studies comparing half-life. All currently available data on the treatment of poisoning with CytoSorb^®^ are summarised in Table 3 and Table 4.

All existing in vivo literature on drugs commonly involved in poisonings is much less informative than that on drugs used to treat sepsis and septic shock. Case reports describing the treatment of poisoning with antidepressants [43,44], antipsychotics [45,47], flecainide [49], and MDMA [13] conclude that the use of CytoSorb^®^ in such cases is reasonable. However, critics point out that differences in half-lives have not been demonstrated, so it cannot be concluded that CytoSorb^®^ actually accelerates the elimination of these drugs [53,54,55]. This has only been clearly shown for mercuric chloride, where it is proven that CytoSorb^®^ shortens the half-life, and consequently, faster clearance of mercuric chloride can be expected [50]. For the other active substances in Table 4, in vitro data are available from two different studies [34,51].

### 2.5. Cardiovascular Surgery

There is a considerable amount of literature on the use of CytoSorb^®^ for drug removal prior to cardiac surgery. However, few studies define pharmacokinetic parameters. Studies demonstrating the removal of anticoagulant and antiplatelet drugs with CytoSorb^®^, from which we were able to calculate percentage removal, are listed in Table 5 and Table 6.

A prospective study of apixaban included patients with acute type A aortic dissection. Eight patients who underwent CytoSorb^®^ during surgery and cardiopulmonary bypass were enrolled. Plasma concentrations of apixaban were shown to decrease with CytoSorb^®^ treatment [56]. A case report by Buncore et al. demonstrates that apixaban was cleared by CytoSorb^®^ at the time of peak plasma concentrations [7]. An in vivo study of ticagrelor was also conducted, which shows that intraoperative use of CytoSorb^®^ with CPB reduced ticagrelor concentrations [57]. All other results presented in Table 6 are from in vitro studies.

### 2.6. Other Indications

Table 7 and Table 8 show the percentage removal and CytoSorb^®^ clearances for drugs that could not be classified in any of the above areas of use. These include immunosuppressants and levosimendan.

Regarding other indications, two studies investigated immunosuppressants, and additional data are available from in vitro studies involving other active substances [34,61,65].

For immunosuppressants, an in vivo study in sheep showed that basiliximab and prednisolone bind minimally to CytoSorb^®^, while tacrolimus, ciclosporin A, mycophenolate mofetil, everolimus, and methylprednisolone bind slightly more. The study provides the maximum binding masses of individual active substances, none of which exceed 5% of the daily dose. Additionally, the pharmacokinetic model demonstrates that CytoSorb^®^ clearance is negligible for all studied drugs [61]. In an ex vivo study, CytoSorb^®^ clearances were compared with the clearance of a single drug during acute or chronic dialysis (simulated dialysis). It was observed that, for some drugs, CytoSorb^®^-associated clearances are higher, leading to decreases in plasma concentrations. However, the authors cautioned that, due to the ex vivo nature of the research, a direct correlation with in vivo conditions is not possible [65].

An in vitro study with levosimendan shows that it binds completely to CytoSorb^®^ within two hours [51].

### 2.7. Clinical Considerations for Drug Dosing

Table 9 presents clinical considerations for the dosage and use of CytoSorb^®^ for selected active substances. Clinical considerations are provided only for drugs with consistent results from in vivo studies of sufficient quality assessed using GRADE recommendations.

## 3. Discussion

Studies conducted on binding of active substances to the CytoSorb^®^ haemoadsorber vary considerably. In addition to outlining the three main areas of CytoSorb^®^ application, they demonstrate the effectiveness of CytoSorb^®^ treatment in various ways [4,6,17,67]. The following section explains the differences between in vitro and in vivo studies, followed by a discussion of CytoSorb^®^’s areas of application, and conclusions regarding the feasibility of future studies.

### 3.1. In Vitro and In Vivo Data

The literature includes studies investigating the binding of active substances to the CytoSorb^®^ haemoadsorber in both in vitro and in vivo settings. Direct transferability from in vitro to in vivo studies has been attempted and appears unsuccessful [12,68]. In vitro models do not account for physiological parameters that affect pharmacokinetics, such as volume of distribution, metabolism, protein binding, and ongoing endogenous mechanisms. In vitro models are usually highly simplified, typically using whole blood, plasma, or albumin solutions for experiments. They do not consider the influence of tissue compartments, organ function, or dynamic changes in drug concentration due to metabolism and excretion [65,69]. For example, Schneider et al. demonstrate that CytoSorb^®^ increased the clearance of some antiinfectives in a porcine model, but the impact on total body clearance was variable and depended on drug properties and time-dependent changes in adsorber efficiency [12]. As in vitro models have a limited volume of distribution (for example, there is no distribution of the active substance into tissues), binding to the haemoadsorber is often related only to the capacity of the adsorber and the flow rate through it. Additionally, the initial concentration is known and often higher because there is no redistribution into tissues. From this perspective, in vitro studies are useful for demonstrating that the active substance binds to CytoSorb^®^, but we cannot infer the in vivo kinetics of binding of active substances to CytoSorb^®^ from in vitro studies. Therefore, it is reasonable to consider that in vitro studies are valuable for designing in vivo research, but for formulating recommendations regarding drug dosage when using CytoSorb^®^, it is important to use data from in vivo studies.

### 3.2. Sepsis and Septic Shock

Sepsis and septic shock remain significant clinical challenges with consistently high mortality rate. Recently, the CytoSorb^®^ haemoadsorber has been used to remove cytokines and other inflammatory mediators [1]. For effective treatment of sepsis and septic shock, the earliest possible administration of an antimicrobial drug is crucial, and it must be given at an appropriate dose to achieve the required pharmacokinetic/pharmacodynamic indexes [70]. This is why the question of antimicrobial drug binding to CytoSorb^®^, and the potential for reduced efficacy of antimicrobial treatment, has arisen in clinical practice. Among antimicrobial drugs, most in vivo studies monitor pharmacokinetic parameters of binding, such as percentage removal and CytoSorb^®^ clearance. The most data are available for vancomycin and meropenem. For meropenem, two in vivo studies are available: one prospective [21] and one retrospective [22]. Both have shown that meropenem clearance due to CytoSorb^®^ is lower than with CRRT; in the prospective study on paediatric patients, it was even negative, implying desorption from the haemoadsorber. A study in a porcine model also shows that meropenem clearance due to CytoSorb is 6.3% higher than endogenous clearance [12]. Similarly, in vitro studies show that meropenem binds insignificantly to the haemoadsorber [35]. The reason the in vitro model predicts the behaviour of meropenem in practice well may be that meropenem has a small volume of distribution and binds to plasma proteins to a very limited extent. Because of the small volume of distribution, the antibiotic is expected to be distributed mainly in the intravascular fluid compartment, resulting in less distribution to other tissues, which closely mimics the in vitro model [68]. In the case of vancomycin, the situation is somewhat different. The in vivo study involving seven patients receiving vancomycin as a continuous infusion shows that vancomycin should be replaced due to binding to the adsorber [19]. Conversely, a case report examining vancomycin binding during both continuous and bolus infusion shows that replacement is necessary only with bolus infusion [18]. In clinical practice, deciding on vancomycin dosing is often easier than with other antibiotics, as TDM for vancomycin is widely available. An in vivo prospective study examining the binding of ceftazidime, amikacin, and levofloxacin in the paediatric population demonstrates that clearance with CytoSorb^®^ increased by 6–12% for amikacin, by 43% for ceftazidime, and by 52–72% for levofloxacin. Nevertheless, CytoSorb^®^ clearance remained lower than CRRT clearance for all tested antibiotics except levofloxacin [21]. Based on the percentage differences alone, it is difficult to determine whether antibiotic doses should be replaced; the authors also note that no significant clinical burden was observed [21]. A retrospective study shows that piperacillin does not bind to CytoSorb^®^, or that clearance is even negative, most likely due to desorption [22]. In the porcine model, clearance of piperacillin increased by 19.4% with CytoSorb^®^ [12]. The authors defined clinically insignificant clearance changes if the difference between endogenous and CytoSorb^®^ clearance was below 30%. Therefore, we can conclude that both studies confirm that the binding of piperacillin to CytoSorb^®^ is not clinically relevant. The study on the porcine model is certainly notable as clinically useful, but its main limitation is high variability of the results [12]. The remaining data are mostly from case reports and in vitro studies, which provide less validity. CytoSorb^®^ haemoadsorption is increasingly used in critically ill patients, and its impact on antibiotic exposure is clinically significant. The extent of drug removal depends on physicochemical properties, protein binding, volume of distribution, and pharmacodynamic targets [68]. Concentration-dependent antibiotics may have lower maximal concentrations or total exposure, while time-dependent agents are particularly susceptible to underdosing because CytoSorb^®^ can reduce the time above the MIC. Drugs with AUC- or trough-driven targets, such as vancomycin and linezolid, may also become subtherapeutic. Augmented renal clearance (ARC) and CRRT further increase clearance, raising the risk of inadequate exposure. Individualised dosing and routine TDM are therefore essential when CytoSorb^®^ is used.

### 3.3. Intoxications

In the treatment of poisoning, the utility of extracorporeal treatments is based upon the premise that the toxicity of a poison correlates with its body burden, and that reducing that burden attenuates toxicity [71]. Case reports describing the treatment of poisoning with CytoSorb^®^ mostly provide concentrations of the active substance before and after CytoSorb^®^ therapy. In this way, we can calculate the percentage removal, which is poorly understood due to changes over time. The percentage removal suggests adsorption of the active substance to the haemoadsorber; nevertheless, the variability of the kinetic profile limits the ability to quantify its clinical relevance. Critiques of the case reports on poisoning with amitriptyline and quetiapine, written by the same author, argue that it is necessary to consider the difference in clearance of the active substance with or without CytoSorb^®^ treatment [53,54]. They claim that only in this way can the effectiveness of the treatment be proven. The correspondents also discuss the extracorporeal treatments in poisoning (EXTRIP) recommendations. The EXTRIP guidelines are a series of evidence-based recommendations developed by the EXTRIP workgroup. These guidelines provide consensus recommendations on the use of extracorporeal therapies—primarily intermittent haemodialysis, haemoperfusion, and CRRT—for the management of specific poisonings. The main criticisms are that the EXTRIP recommendations do not advise extracorporeal treatment for the agents mentioned. The primary criteria of the EXTRIP recommendations are low molecular weight, low protein binding, small volume of distribution, and water solubility. All agents described in the case reports (amitriptyline, quetiapine, MDMA, clozapine, imipramine, flecainide) are highly protein bound and have a large volume of distribution, which the EXTRIP recommendations consider unsuitable for extracorporeal treatment [13,44,45,47,49,53,71]. If we consider these critiques and compare the half-life with and without CytoSorb^®^ treatment, we can speculate that for amitriptyline and MDMA, the reduction in half-life achieved with CytoSorb^®^ is too modest to meaningfully support its use in these cases of intoxication [13,43]. Additionally, several case reports describe poisonings with various active substances (patent blue, mercuric chloride, venlafaxine, amlodipine, quetiapine, Montivipera xanthina), but they do not include measurements of active substance concentrations before and after CytoSorb^®^ or other pharmacokinetic parameters that would allow inclusion in our systematic review [14,72,73,74,75,76,77]. In vitro experiments on the removal of active substances in cases of poisoning provide limited information about clinical use, as already discussed in more detail above [34,51].

### 3.4. Cardiovascular Surgery

Removal of anticoagulant and antiplatelet drugs is particularly important to reduce the risk of bleeding if a patient requires emergency surgery. In cardiac surgery, numerous studies have examined the removal of anticoagulant and antiplatelet drugs using CytoSorb^®^. Intraoperative antithrombotic drug removal by haemoadsorption is a novel strategy to reduce perioperative bleeding in patients on antithrombotic therapy undergoing cardiac surgery. The international Safe and Timely Antithrombotic Removal (STAR) registry reports real-world clinical outcomes associated with this approach. Bleeding complications, 24 h chest tube drainage, blood product transfusion, re-operation for bleeding, and in-hospital mortality are included in the STAR registry [78]. The results of the systematic review presented in Table 3 indicate that there is limited data on the binding of anticoagulants and antiplatelet drugs to haemoadsorbers. Specifically, only three in vivo studies have been conducted in which concentrations were measured before and after haemoadsorption with CytoSorb^®^ [7,56,57]. Several studies have measured anti-Xa levels, the value of which cannot be directly correlated with the concentration and clinical effect of the anticoagulant. However, it is known that elevated anti-Xa levels increase the risk of bleeding [56,79]. Nevertheless, several studies demonstrate that the use of CytoSorb^®^ before and during cardiac surgery is feasible and safe [6,17,79,80,81,82,83,84,85,86]. Given that cardiac surgery is an emergency requiring prompt intervention, it is questionable whether studies including percentage removal are necessary or provide any additional information beyond the data included in the STAR registry. It is also important to consider that the antidote andexanet alfa (for rivaroxaban and apixaban) is significantly more expensive than haemoadsorption with CytoSorb^®^ [87], and that an antidote for edoxaban has not yet been officially approved [88]. This has likely prompted research into alternative methods for removing antithrombotic drugs prior to cardiac surgery. CytoSorb^®^ is now CE mark-approved for the intraoperative removal of ticagrelor and rivaroxaban during on-pump cardiac surgery [86]. The in vitro study on apixaban clearly shows that apixaban is more effectively removed at higher concentrations [89], which could be advantageous in the treatment of anticoagulant poisoning. In our view, direct transfer is not possible, as CytoSorb^®^ flows in CPB circuits are typically higher (300 mL/min) than flows during simultaneous use with dialysis methods (150–200 mL/min). A randomised clinical trial (TORNADOEs) is being conducted to assess the removal of direct anticoagulants with CytoSorb^®^, in which the primary endpoints are plasma anticoagulant concentrations and the incidence of bleeding [7].

### 3.5. Other Indications

In the area of other active ingredients, most data are available on immunosuppressants. Two studies were conducted: one in vivo on a sheep model and the other ex vivo [61,65]. Both studies aimed to investigate immunosuppressant use in the context of organ transplantation, where a common complication is a pronounced inflammatory response resulting from ischaemia–reperfusion injury and resultant cytokine release, which may trigger a systemic inflammatory response. For this reason, patients after transplantation are often connected to haemoadsorption with CytoSorb^®^, and the removal of immunosuppressants can lead to rejection of the transplanted organ. In vivo data on sheep showed that less than 5% of the daily dose is bound for all tested immunosuppressants, which is considered clinically insignificant. The authors specifically emphasised that in vitro data cannot be directly transferred to an in vivo setting, that overall pharmacokinetics change in critically ill patients, which is not accounted for in their sheep model, and that adsorption does not always reduce the concentrations of active substances below the therapeutic range [61]. The ex vivo study was somewhat contradictory, as it shows that methylprednisolone, mycophenolate, 6-mercaptopurine and cyclosporin bind significantly to CytoSorb^®^, but tacrolimus does not. This is explained by the strong binding of tacrolimus to plasma proteins. The study also compared the removal of selected immunosuppressants by CytoSorb^®^ with that of acute and chronic haemodialysis. Only methylprednisolone and 6-mercaptopurine were significantly removed by dialysis, and it is known that drugs strongly bound to plasma proteins are poorly dialyzed [65]. For levosimendan, there is only one in vitro study, which is difficult to rely on [51].

### 3.6. Recommendations for Future Research

Based on the systematic review of the literature, we can draw several conclusions regarding the feasibility of further research into the binding of active substances to the CytoSorb^®^ haemoadsorber. Our literature review highlights the importance of distinguishing between in vitro and in vivo research. We have demonstrated that in vitro findings are not directly transferable to in vivo settings; while in vitro studies can determine whether an active substance binds to the haemoadsorber, in vivo research is necessary to provide clinically relevant information. As patients with sepsis and septic shock have unique pharmacokinetic and pharmacodynamic profiles, it is essential that studies on the binding of antibiotics, antifungals, and antivirals are conducted specifically in these patient groups. Studies that compare the clearance achieved by CytoSorb^®^ with other forms of clearance—most commonly endogenous clearance or clearance by dialysis—provide the most informative data. This information is crucial in determining whether an active substance should be replaced during haemoadsorption. It would be reasonable to conduct similarly designed studies in the field of immunosuppressant use. In cases of intoxication, where a single dose of an active substance needs to be removed from the body as quickly as possible, the adsorption capacity of the haemoadsorber is of key importance. However, studies have shown that the half-life of many active substances did not decrease despite the use of CytoSorb^®^. To facilitate practical application, it would be beneficial to demonstrate the adsorption capacity and the time at which this is achieved, as it is known that the adsorber can reach saturation relatively quickly in a clinical setting [90]. This would provide information on when the haemoadsorber should be replaced. In cardiac surgery, the situation is somewhat different. Although pharmacokinetic parameters have been shown to be important for the use of CytoSorb^®^ in sepsis and intoxication, they do not appear to be as significant when used before and during open-heart cardiac surgery. The parameters defined in the STAR registry seem robust enough to indicate whether the haemoadsorption method is appropriate and safe for a particular antithrombotic agent. It is also important to note that CytoSorb^®^ is combined with various extracorporeal systems, such as CRRT, ECMO, and CPB. The flow rates through these systems differ, and consequently, the binding kinetics also vary. The systematic review clearly shows heterogeneity in the presentation of results, which further complicates the transfer of knowledge about binding into practice. It should be noted that binding changes over time, so it is reasonable to determine when binding is at its maximum and how it evolves. In the future, it is reasonable to adopt recommendations for implementation in research on the binding of active substances to the CytoSorb^®^ haemoadsorber and to unify procedures, as has already been done in the STAR registry.

### 3.7. Clinical Significance of the Results

From a pharmacokinetic/pharmacodynamic (PK/PD) perspective, the clinical relevance of CytoSorb^®^ drug removal depends on how adsorption alters systemic exposure relative to the drug’s PK/PD target (e.g., Cmax/MIC, AUC/MIC, fT > MIC, or trough concentrations). Adsorption should therefore be considered not only in terms of instantaneous extraction from plasma, but also in the context of total body clearance, distribution, dosing regimen, and the specific PK/PD index that best predicts efficacy or toxicity. The extent of drug elimination during CytoSorb^®^ haemoadsorption is often expressed as “percentage removal,” which reflects the proportion of the drug cleared from the plasma compartment over a defined period. While useful for characterising the instantaneous efficiency of the adsorber, percentage removal does not indicate the total amount of the drug eliminated from the body, nor does it directly predict pharmacodynamic consequences. After the initial decline in plasma levels, many drugs redistribute from peripheral tissues back into the intravascular space, partially restoring plasma concentrations and providing an additional substrate for adsorption. This redistribution is particularly relevant for drugs with a large volume of distribution, where equilibration is slow and removal remains limited. CytoSorb^®^ primarily adsorbs the unbound fraction of a drug; however, as a free drug is removed, the protein-bound drug dissociates to re-establish equilibrium, enabling continued adsorption until binding sites or the adsorber itself become saturated. Saturation of the haemoadsorber reduces adsorption efficiency over time and may, under certain conditions, lead to desorption. As adsorption is a reversible process, a steep decline in plasma concentration or competitive displacement by other molecules can shift the equilibrium, allowing a previously adsorbed drug to re-enter circulation. These dynamic interactions highlight that drug removal by CytoSorb^®^ is governed by complex pharmacokinetic and physicochemical principles, and that percentage removal alone cannot fully describe the clinical relevance of adsorption. When CytoSorb^®^ clearance is quantified (for example, as an extracorporeal clearance value) and compared with endogenous clearance or known elimination half-lives, the impact on overall drug exposure can be more meaningfully assessed. If CytoSorb^®^ clearance is small compared to endogenous clearance, the effect on total body clearance and area under the curve (AUC) is likely to be modest; conversely, when CytoSorb^®^ provides clearance of similar or greater magnitude, substantial reductions in exposure and a shortened half-life can be expected, particularly during the active adsorption phase. In critically ill patients, additional extracorporeal techniques such as CRRT further alter drug disposition by introducing another clearance pathway. For hydrophilic, low-protein-bound drugs with small volumes of distribution, the combined effect of CRRT and CytoSorb^®^ can significantly increase total clearance and reduce systemic exposure, jeopardising achievement of PK/PD targets if dosing is not adjusted. Therefore, the interpretation of CytoSorb^®^-mediated adsorption should include percentage removal, device-specific clearance, endogenous elimination, and any concurrent extracorporeal therapies within a PK/PD framework, with dose individualisation and, where feasible, therapeutic drug monitoring as essential tools to maintain effective and safe drug exposure.

### 3.8. Limitations and Strenghts

This systematic review has several limitations. The available evidence on drug adsorption by the CytoSorb^®^ haemoadsorber is limited, resulting in a small number of eligible studies. To capture all relevant information, we included in vitro and in vivo studies, both on animals and humans, which differ markedly in methodological quality and clinical relevance. This heterogeneity restricted comparability and made quantitative synthesis difficult. Methodological variability was most pronounced in the in vitro studies, where substantial differences existed in experimental media (plasma vs. whole blood), volume-to-sorbent ratios and flow conditions. Some studies used suboptimal or non-standardised approaches, reducing reproducibility. Animal studies were often limited by small sample sizes, species differences, and incomplete reporting of key design features. The human studies were observational and heterogeneous regarding patient characteristics, co-therapies, and illness severity. Several did not clearly distinguish adsorption by CytoSorb^®^ from dialysis-related or endogenous clearance or other pharmacokinetic parameters, limiting interpretation. Consequently, obtained data did not allow clinically meaningful dosing guidance. Reporting quality varied across studies, with some results available only as graphs, requiring digital extraction thus contributing to additional variability. Finally, our systematic review protocol was not registered; however, all methodological steps adhered to PRISMA 2020 recommendations. Despite these limitations, the review provides a structured synthesis of the current evidence and offers preliminary dosing considerations for selected active substances based on the most robust data available.

## 4. Materials and Methods

### 4.1. Design

A systematic review was conducted following the Preferred Reporting Items for Systematic Reviews and Meta-Analyses (PRISMA) guidelines [91]; however, the review protocol was not registered in PROSPERO or any other registry. PRISMA 2020 for Abstract Checklist and PRISMA 2020 Checklist are available in the Supplementary Materials (Tables S1 and S2).

### 4.2. Search Strategy

A comprehensive systematic literature search was performed in PubMed and Ovid MEDLINE from the database inception to 31 December 2025 by two independent researchers (S.K and T.V.). The search strategy included the “cytosorb” and its variations (e.g., “Cytosorb”, “CytoSorb”), using appropriate Boolean operators. References of previously published reviews focusing on drug adsorption to CytoSorb^®^ were manually searched for any additional records. The full search strategy is available in the Supplementary Materials (Tables S7 and S8).

### 4.3. Inclusion and Exclusion Criteria

The search was based on the research question PICO (Patients, Interventions, Comparison and Outcomes) [91].

This review covers studies that investigate drug adsorption to CytoSorb^®^. Studies were grouped based on CytoSorb^®^ application: (i) treatment of sepsis and septic shock, (ii) treatment of intoxications, (iii) removal of drugs before cardiac surgery, and (iv) other indications. Both in vivo and in vitro data were included, but the research had to be conducted at least on blood, not on albumin solutions or other non-blood substitutes. Articles that reported or enabled calculation of percentage removal and those that defined CytoSorb^®^ clearance/half-life compared to clearance/half-life without CytoSorb^®^ were included. If we could not extract percentage removal directly from the article, we calculated it from the available data using the following formula:

$$\%removal=\frac{C_{\mathrm{pre}}-C_{\mathrm{post}}}{C_{\mathrm{pre}}}\times 100$$

C_pre_, concentration before CytoSorb^®^ (maximal concentration),

C_post_, concentration after CytoSorb^®^ (minimal concentration).

Conference abstracts were not considered because full texts were not available.

We report data as they are most commonly presented by the original studies. For the in vitro studies, maximal percentage removal (max. % removal) was calculated from the starting concentration (Cpre) and the lowest concentration during the adsorption experiment (Cpost), i.e., the % removal for the entire in vitro haemoadsorption procedure. For the in vivo studies, the percentage removal refers to measurements taken from the circuit before and after haemoadsorber cartridge, i.e., instantaneous removal, at given time points, and a range of values was calculated and reported together with respective time points, to reflect the adsorber saturation.

### 4.4. Study Selection and Data Extraction

Two independent reviewers (S.K. and T.V.) conducted the initial search in which the total number of records identified in the search was calculated. The total number of records screened was noted, in addition to the deleted records, after reading the title and abstract. Once the duplicates had been eliminated with Zotero^®^, a selection by title and abstract was made before the manuscripts were read in full. Those studies that met the eligibility criteria were included in this review. If there was any debate, a consensus was reached with the third author (J.G.).

Finally, the same authors extracted the following information from the included studies: first author; title; year of publication; active substance; clinical considerations/conclusions; study type; a brief description of the study (in vivo): patient/animal population (sepsis vs. intoxications vs. cardiac surgery vs. other); extracorporeal modality (CRRT, CPB, ECMO); pharmacokinetic endpoints (percentage removal, clearance, half-life); blood flow rate or a brief description of the model (in vitro): extracorporeal modality (CRRT, CPB, ECMO); pharmacokinetic endpoints (percentage removal, clearance, half-life); blood flow rate; blood volume; CytoSorb^®^ volume. In cases where it was necessary to calculate individual parameters, the concentrations were exported to Excel. Data provided only in graphical format were extracted with Digit software version 1.0.4.

### 4.5. Assessment of Methodological Quality

Two independent reviewers (S.K. and T.V.) assessed the quality and methodological validity of the selected studies. Due to the wide variety of study types, four different criteria were used. We assessed the quality of human observational cohort studies using the Newcastle–Ottawa Scale (NOS) [92]. For animal studies, we used the SYRCLE risk of bias tool [93]. We applied the JBI critical appraisal checklist for case reports [94], and the ToxRTool scorecard [95] for in vitro studies.

For each reviewed study, we also assessed the quality of evidence according to the Grades of Recommendation, Assessment, Development, and Evaluation (GRADE) Handbook [96]. We found no studies with high-quality evidence. We included observational in vivo studies that compared CytoSorb^®^ clearance with clearance without CytoSorb^®^ in the moderate category. Observational studies that did not compare CytoSorb^®^ clearance with clearance without CytoSorb^®^ were placed in the low category. We included all case reports and all in vitro studies in the very low category.

### 4.6. Data Synthesis

Results are reported descriptively. Meta-analysis was not applicable due to heterogeneity of studies.

## 5. Conclusions

The number of studies on drug binding to the CytoSorb^®^ haemoadsorber is increasing each year. However, in vivo studies providing clinically useful data remain limited. It would be reasonable to establish a methodology for conducting studies on the binding of active substances to the haemoadsorber according to the areas of use, and, where possible, to provide TDM monitoring of the drugs.

## Figures and Tables

**Table 1:** Summary of in vivo evidence on drug binding to CytoSorb^®^ for drugs used in sepsis and septic shock.

Active Substance (V_D_ (L), fb (%))	Instantaneous % Removal (Range (Time))	CL Cyto (L/h)	CL (Endo, CRRT) (L/h)	EC Modality/BFR (mL/min)	Summary (Source Defined)	Study Type	Level of Evidence	Ref
Antibiotics—glycopeptides								
teicoplanin (V_D_ (56–112) °; fb (87–97))	6 (360 min), 48 (15 min)	-	-	HP/200	concentrations after 6 h were within the therapeutic range.	case report	very low	[18]
	*	1.9	CL_endo_: 5.6	HP/150–200	significant CL_cyto_	in vivo on pigs	low	[12]
vancomycin (V_D_ (28–70) °; fb (50))	8, 24 (within one CytoSorb^®^ treatment)	6. 7	CL_endo_: 2.3	CRRT/100	need for additional 500 mg in first 2 h.	in vivo, prospective + pharmacokinetic modelling	low/moderate	[19]
	3 (240 min), 98 (15 min)—bolus infusion, 4 (480 min), 60 (15 min)—continuous infusion	-	-	HP/200	concentrations are below the therapeutic range with bolus infusion (after 15 min), but within the therapeutic range with continuous infusion.	case report	very low	[18]
Antibiotics—beta-lactams								
cefepime (V_D_ (18); fb (20))	*	0	CL_endo_: 1.3	HP/150–200	insignificant CL_cyto_	in vivo on pigs	low	[12]
cefiderocol (V_D_ (18); fb (40–60))	^+^ (1440 min), 58 (180 min)	−1.1, 3.4	CL_CRRT_: 1.0, 3.1	CRRT/-	additional dose in first 2 h	case report	very low	[20]
ceftazidime (V_D_ (15–20); fb (5–22.8))	20 (within dosing interval)	1.5	CL_CRRT_: 2	CRRT/125	low impact on removal	in vivo, prospective	low	[21]
ceftriaxone (V_D_ (4.48–35.2); fb (95))	*	0.7	CL_endo_: 10.8	HP/150–200	insignificant CL_cyto_	in vivo on pigs	low	[12]
flucloxacillin (V_D_ (12.6–13.3) °; fb (95–96))	*	1.5	CL_endo_: 9.8	HP/150–200	insignificant CL_cyto_	in vivo on pigs	low	[12]
meropenem (V_D_ (17.5) °; fb (2))	#	−1.5	CL_CRRT_: 1.1	CRRT/125	negative impact on removal	in vivo, prospective	low	[21]
	1 (within dosing interval)	0	CL_CRRT_: 2.4	CRRT/200	did not identify removal	in vivo, retrospective	low	[22]
	*	1.7	CL_endo_: 24	HP/150–200	insignificant CL_cyto_	in vivo on pigs	low	[12]
piperacillin (V_D_ (17) °; fb (30))	#	−0.6	CL_CRRT_: 3.4	CRRT/200	did not identify removal	in vivo, retrospective	low	[22]
	*	1.9	CL_endo_: 9.6	HP/150–200	insignificant CL_cyto_	in vivo on pigs	low	[12]
Antibiotics—fluoroquinolones								
ciprofloxacin (V_D_ (140–210) °; fb (20–40))	*	1.9	CL_endo_: 11.9	HP/150–200	insignificant CL_cyto_	in vivo on pigs	low	[12]
levofloxacin (V_D_ (89–112); fb (24–38))	C_max_: 63; C_min_: 25 (within dosing interval)	C_max_: 4.7; C_min_: 1.9	CL_CRRT_; C_max_: 1.8; C_min_: 1.7	CRRT/125	some impact on removal	in vivo, prospective	low	[21]
Antibiotics—aminoglycosides								
amikacin (V_D_ (24); fb (10))	C_max_: 3; C_min_: 1 (within dosing interval)	C_max_: 0.2; C_min_: 0.1	CL_CRRT_: 1.5 (C_max_ and C_min_)	CRRT/125	low impact on removal	in vivo, prospective	low	[21]
tobramycin (V_D_ (14–24.5) °; fb (<10))	*	0.5	CL_endo_: 8.1	HP/150–200	insignificant CL_cyto_	in vivo on pigs	low	[12]
Antibiotics—others								
clarithromycin (V_D_ (140–210) °; fb (70–75))	*	3.7	CL_endo_: 65	HP/150–200	insignificant CL_cyto_	in vivo on pigs	low	[12]
clindamycin (V_D_ (43–74); fb (60–94))	*	4.1	CL_endo_: 56	HP/150–200	insignificant CL_cyto_	in vivo on pigs	low	[12]
linezolid (V_D_ (40–50); fb (31))	*	4.6	CL_endo_: 3.9	HP/150–200	significant CL_cyto_	in vivo on pigs	low	[12]
	34 (480 min), 100 (60 min)	-	-	CRRT/200	concentrations are below the therapeutic range. Need for TDM at higher doses.	case report	very low	[23]
metronidazole (V_D_ (35.7–77) °; fb (20))	*	0.2	CL_endo_: 1.0	HP/150–200	insignificant CL_cyto_	in vivo on pigs	low	[12]
Antifungals								
anidulafungin (V_D_ (30–50); fb (84))	*	0.7	CL_endo_: 2.2	HP/150–200	insignificant CL_cyto_	in vivo on pigs	low	[12]
fluconazole (V_D_ (39); fb (11–12))	*	4.0	CL_endo_: 1.3	HP/150–200	significant CL_cyto_	in vivo on pigs	low	[12]
liposomal amphotericin B (V_D_ (29.4–53.9)°; fb (>95))	*	2.1	CL_endo_: 2.5	HP/150–200	significant CL_cyto_	in vivo on pigs	low	[12]
posaconazole (V_D_ (1774); fb (>98))	*	4.1	CL_endo_: 13	HP/150–200	significant CL_cyto_	in vivo on pigs	low	[12]
Antivirals								
ganciclovir (V_D_ (51.8) °; fb (1–2))	*	−0.1	CL_endo_: 7.9	HP/150–200	insignificant CL_cyto_	in vivo on pigs	low	[12]

° V_D_ originally given in units of L/kg and recalculated for a typical patient weighing 70 kg. * Inconsistent data due to high variability. ^+^ Inconsistent data due to unclear presentation of the results. # Negative values reported, but likely clinically insignificant. Abbreviations: V_D_, volume of distribution; fb, fraction bound; max. % removal, maximal percentage removal; CL_Cyto_, CytoSorb^®^ clearance; CL_endo_, endogenous clearance; CL_CRRT_, CRRT clearance; C_max_, maximal concentration; C_min_, minimal concentration; EC, extracorporeal; HP, haemoperfusion; CRRT, continuous renal replacement therapy; BFR, blood flow rate; Ref, reference. References for V_D_ and fb: teicoplanin [24]; vancomycin, cefepime, cefiderocol, ceftazidime, ceftriaxone, ciprofloxacin, levofloxacin, amikacin, clindamycin, linezolid, metronidazole, anidulafungin, fluconazole, posaconazole, ganciclovir [25]; flucloxacillin [26]; meropenem [27]; piperacillin [28]; tobramycin [29,30]; clarithromycin [31]; liposomal amphotericin B [32,33].

**Table 2:** Summary of in vitro evidence on drug binding to CytoSorb^®^ for drugs used in sepsis and septic shock.

Active Substance (V_D_ (L), fb (%))	Max % Removal (Time)	CL Cyto (L/h)	CL (Endo, CRRT) (L/h)		EC Modality/BFR (mL/min)	Summary (Source Defined)	Level of Evidence	Ref
	Antibiotics—glycopeptides							
teicoplanin (V_D_ (56–112) °; fb (87–97))	95 (120 min)	-	-		HP/250	-	very low	[34]
vancomycin (V_D_ (28–70) °; fb (50))	94 (120 min)	-	-		HP/250	-	very low	[34]
	Antibiotics—beta-lactams							
meropenem (V_D_ (17.5) °; fb (2))	-	1.4, 5.4	CL_CRRT_: 2		CRRT/200	During 18 h, 384 mg were adsorbed.	very low	[35]
	Antibiotics—fluoroquinolones							
ciprofloxacin (V_D_ (140–210) °; fb (20–40))	-	4.9, 6.3	CL_CRRT_: 1.7		CRRT/200	During 18 h, 284 mg were adsorbed.	very low	[35]
	Antibiotics—aminoglycosides							
amikacin (V_D_ (24); fb (10))	12 (15 min)	-	-		HP/250	-	very low	[34]
gentamicin (V_D_ (14–28) °; fb (0–30))	28 (30 and 120 min)	-	-		HP/250	-	very low	[34]
netilmicin (V_D_ (17.5–47.6) °; fb (<10))	33 (15 and 120 min)	-	-		HP/250	-	very low	[34]
tobramycin (V_D_ (14–24.5) °; fb (<10))	22 (30 min)	-	-		HP/250	-	very low	[34]
	Antivirals							
remdesivir (V_D_ (18–20); fb (88–93))	100 (60 min)	-	-		HP/200	-	very low	[36]

° V_D_ originally given in units of L/kg and recalculated for a typical patient weighing 70 kg. Abbreviations: V_D_, volume of distribution; fb, fraction bound; max. % removal, maximal percentage removal; CL_Cyto_, CytoSorb^®^ clearance; CL_endo_, endogenous clearance; CL_CRRT_, CRRT clearance; C_max_, maximal concentration; C_min_, minimal concentration; EC, extracorporeal; HP, haemoperfusion; CRRT, continuous renal replacement therapy; BFR, blood flow rate; Ref, reference. References for V_D_ and fb: teicoplanin [24]; vancomycin, ciprofloxacin, amikacin [25]; meropenem [27]; gentamicin [37]; netilmicin [38]; tobramycin [29,30]; remdesivir [39].

**Table 3:** Summary of in vivo evidence on drug binding to CytoSorb^®^ for drugs used in cases of intoxication.

Active Substance (V_D_ (L), fb (%))	Instantaneous % Removal (Range (Time))	t_1/2_ Cyto (h, min)	t_1/2_ (Endo, CRRT) (h, min)	EC Modality/BFR (mL/min)	Summary (Source Defined)	Study or Article Type	Level of Evidence	Ref
Antidepressants								
amitriptyline (V_D_ (769–1702); fb (95))	78 (480 min), 98 (at the beginning of therapy)	22 h (case 1), 8.5 h (case 2)	t_1/2endo_: 12.9–36.1 h [41,42]	CRRT/100	-	case report	very low	[43]
imipramine (V_D_ (700–1400) °; fb (60–96))	12, 90 (time NA)	-	-	CRRT/-	-	case report	very low	[44]
Antipsychotics								
clozapine (V_D_ (272–1290); fb (97))	0 (660 min and 1218 min), 84 (at the beginning of therapy)	-	-	CRRT/-	-	case report	very low	[45]
quetiapine (V_D_ (420–980) °; fb (83))	5 (1800 min), 79 (120 min)	-	t_1/2endo_: 7 h [46]	CRRT/100	-	case report	very low	[47]
Other								
digoxin (V_D_ (475–500); fb (25))	18 (1404 min), 79 (30 min)	-	-	CRRT/-	-	case report	very low	[48]
flecainide (V_D_ (350–938) °; fb (40))	12 (270 min), 67 (15 min)	-	-	CRRT/200	-	case report	very low	[49]
mercuric chloride (V_D_ (NA); fb (NA))	-	t_1/2CRRT+Cyto_: 19.3 h—phase I elimination; 86.6 h—phase II elimination	t_1/2CRRT_: 40.8 h—phase I elimination; 115.5 h—phase II elimination	CRRT/150 −200	CytoSorb^®^ is usefull in acute poisoning with mercuric chloride	case report	very low	[50]

° V_D_ originally given in units of L/kg and recalculated for a typical patient weighing 70 kg. Abbreviations: V_D_, volume of distribution; fb, fraction bound; max. % removal, maximal percentage removal; t_1/2Cyto_, CytoSorb^®^ half-life; t_1/2endo_, endogenous half-life; t_1/2CRRT+Cyto_, CRRT and CytoSorb^®^ half-life; EC, extracorporeal; CRRT, continuous renal replacement therapy; BFR, blood flow rate; Ref, reference; NA, not available. References for V_D_ and fb: amitriptyline, imipramine, clozapine, quetiapine, flecainide, digoxin [25].

**Table 4:** Summary of in vitro evidence on drug binding to CytoSorb^®^ for drugs used in cases of intoxication.

Active Substance (V_D_ (L), fb (%))	Max % Removal (Time)	EC/and BFR (mL/min)	Summary (Source Defined)	Level of Evidence	Ref
Antiepileptics					
carbamazepine (V_D_ (49–98) °; fb (75–80))	76 (60 min)	HP/40 (CytoSorb^®^: 60 mL)	-	very low	[51]
lamotrigine (V_D_ (63–91) °; fb (55))	94 (5 min)	HP/40 (CytoSorb^®^: 60 mL)	-	very low	[51]
oxcarbazepine (V_D_ (49); fb (40))	100 (1 min)	HP/40 (CytoSorb^®^: 60 mL)	-	very low	[51]
phenobarbital (V_D_ (42–54.6) °; fb (40–45))	98 (120 min)	HP/250	-	very low	[34]
phenytoin (V_D_ (52.5) °; fb (90))	98 (5 min)	HP/40 (CytoSorb^®^: 60 mL)	-	very low	[51]
	100 (60 min)	HP/250	-	very low	[34]
valproate (V_D_ (19); fb (81.5–90))	51 (5 min)	HP/40 (CytoSorb^®^: 60 mL)	-	very low	[51]
	81 (120 min)	HP/250	-	very low	[34]
Other					
digoxin (V_D_ (475–500); fb (25))	100 (120 min)	HP/250	-	very low	[34]
MDMA (V_D_ (NA); fb (NA))	100 (5 min)	CRRT/80	-	very low	[13]
metformin (V_D_ (296–1012); fb (negligible))	29 (300 min)	HP/40 (CytoSorb^®^: 60 mL)	-	very low	[51]
methylene blue (V_D_ (NA); fb (NA))	99 (15 min)	HP/40 (CytoSorb^®^: 60 mL)	-	very low	[51]
theophylline (V_D_ (21–49) °; fb (40))	74 (15–60 min)	HP/250	-	very low	[34]

° V_D_ originally given in units of L/kg and recalculated for a typical patient weighing 70 kg. Abbreviations: V_D_, volume of distribution; fb, fraction bound; max. % removal, maximal percentage removal; t_1/2Cyto_, CytoSorb^®^ half-life; t_1/2endo_, endogenous half-life; t_1/2CRRT+Cyto_, CRRT and CytoSorb^®^ half-life; EC, extracorporeal; HP, haemoperfusion; CRRT, continuous renal replacement therapy; BFR, blood flow rate; Ref, reference; NA, not available. References for V_D_ and fb: carbamazepine, lamotrigine, oxcarbazepine, valproate, digoxin, metformin, theophylline [25]; phenobarbital [52].

**Table 5:** Summary of in vivo evidence on drug binding to CytoSorb^®^ for drugs used in cardiovascular surgery.

Active Substance (V_D_ (L), fb (%))	Instantaneous % Removal (Range (Time))	EC Modality/BFR (mL/min)	Summary (Source Defined)	Study Type	Level of Evidence	Ref.
Anticoagulants						
apixaban (V_D_ (21); fb (92–94))	41 (5 min), 63 (90 min)	CPB/-	Haemoadsorption is feasible and safe.	prospective cohort study	low	[56]
	21 (300 min), 78 (15 min)	CPB/-	CytoSorb may increase apixaban clearance and might facilitate perioperative haemostatic management.	case report	very low	[7]
Antiplatelets						
ticagrelor (V_D_ (88); fb (>99))	67 (within one CytoSorb^®^ treatment)	CPB/450–550	Intraoperative haemoadsorption can efficiently remove ticagrelor.	in vivo	low	[57]

Abbreviations: V_D_, volume of distribution; fb, fraction bound; max. % removal, maximal percentage removal; EC, extracorporeal; BFR, blood flow rate; Ref, reference; NA, not available. References for V_D_ and fb: apixaban, ticagrelor [25].

**Table 6:** Summary of in vitro evidence on drug binding to CytoSorb^®^ for drugs used in cardiovascular surgery.

Active Substance (V_D_ (L), fb (%))	Max % Removal (Time)	EC Modality/BFR (mL/min)	Level of Evidence	Ref.
Anticoagulants				
apixaban (V_D_ (21); fb (92–94))	100 (30 min)	HP/40 (CytoSorb^®^: 60 mL)	very low	[51]
argatroban (V_D_ (12.18); fb (54))	87 (60 min)	HP/40 (CytoSorb^®^: 60 mL)	very low	[51]
edoxaban (V_D_ (107); fb (55))	98 (time NA)	HP/-(CytoSorb^®^: 40 mL)	very low	[58]
rivaroxaban (V_D_ (50); fb (92–95))	92 (120 min)	HP/40 (CytoSorb^®^: 60 mL)	very low	[59]
Antiplatelets				
ticagrelor (V_D_ (88); fb (>99))	>99 (180 min)	HP/9 (CytoSorb^®^: 10 mL); 17 (CytoSorb^®^: 300 mL); 3 (CytoSorb^®^: 10 mL)	very low	[60]

Abbreviations: V_D_, volume of distribution; fb, fraction bound; max. % removal, maximal percentage removal; EC, extracorporeal; HP, haemoperfusion; BFR, blood flow rate; Ref, reference; NA, not available. References for V_D_ and fb: apixaban, argatroban, edoxaban, rivaroxaban, ticagrelor [25].

**Table 7:** Summary of in vivo evidence on immunosuppressive agents and levosimendan binding to CytoSorb^®^.

Active Substance (V_D_ (L), fb (%))	Instantaneous % Removal (Range (Time))	CLcyto (L/h)	EC Modality/BFR (mL/min)	Summary (Source Defined)	Study Type	Level of Evidence	Ref.
Immunosuppressants							
basiliximab (V_D_ (8–9.7); fb (NA))	0 ^$^ (150 min), 9 (30 min)	0.1, 0.4	HP/120	lack of removal	in vivo on sheep	low	[61]
cyclosporin A (V_D_ (280–560) °; fb (90))	2 (0 min), 27 (30 min)	0.5, 1.6	HP/120	very limited influence on blood levels	in vivo on sheep	low	[61]
everolimus (V_D_ (350–630) °; fb (74))	0 ^$^ (180 min), 22 (30 min)	0.1, 0.7	HP/120	very limited influence on blood levels	in vivo on sheep	low	[61]
methylprednisolone (V_D_ (96.6) °; fb (76.8))	#	#	HP/120	very limited influence on blood levels	in vivo on sheep	low	[61]
mycophenolate mofetil (V_D_ (147–364) °; fb (97))	0.4 (0 min), 57 (30 min)	0.2, 2.6 ^+^	HP/120	very limited influence on blood levels	in vivo on sheep	low	[61]
prednisolone (V_D_ (29.3–44.2); fb (65–91))	*	−0.8, 0.7 ^+^	HP/120	lack of removal	in vivo on sheep	low	[61]
tacrolimus (V_D_ (60.9–343) °; fb (99))	0 ^$^ (180 min), 38 (30 min)	0.2, 1.3	HP/120	very limited influence on blood levels	in vivo on sheep	low	[61]

° V_D_ originally given in units of L/kg and recalculated for a typical patient weighing 70 kg. * Inconsistent data due to high variability. ^$^ Negative values reported, but they were not clinically significant and were likely the result of imprecision when extracting concentration values from the graphical data. ^+^ Result from experiments in three different combinations. # Inconsistent data due to different dosing regimens. Abbreviations: V_D_, volume of distribution; fb, fraction bound; max. % removal, maximal percentage removal; CL_Cyto_, CytoSorb^®^ clearance; CL_AD_, acute dialysis clearance; CL_CD_, chronic dialysis clearance; EC, extracorporeal; HP, haemoperfusion; BFR, blood flow rate; Ref, reference; NA, not available. References for V_D_ and fb: basiliximab [62]; cyclosporine A, methylprednisolone, mycophenolate mofetil, prednisolone, tacrolimus [25]; everolimus [63]; 6-mercaptopurine [64].

**Table 8:** Summary of in vitro evidence on immunosuppressive agents and levosimendan binding to CytoSorb^®^.

Active Substance (V_D_ (L), fb (%))	Max % Removal (Time)	CLcyto (L/h)	CL (AD, CD) (L/h)	EC Modality/BFR (mL/min)	Study Type	Level of Evidence	Ref.
Immunosuppressants							
cyclosporin A (V_D_ (280–560) °; fb (90))	32 (240 min)	0.09, 0.2	CL_AD_: −0.02, 0.6; CL_CD_: 0.02, 1.4	HP/30 (CytoSorb^®^: 30 mL)	ex vivo	very low	[65]
	47 (120 min)	-	-	HP/250	in vitro	very low	[34]
6-mercaptopurine (V_D_ (63) °; fb (19))	47 (240 min)	0.3, 1.8	CL_AD_: 0.4, 2.0; CL_CD_: 1.0, 11.3	HP/30 (CytoSorb^®^: 30 mL)	ex vivo	very low	[65]
methylprednisolone (V_D_ (96.6) °; fb (76.8))	92 (180 min)	1.0, 2.9	CL_AD_: 0.2, 1.7; CL_CD_: 1.2, 4.5	HP/30 (CytoSorb^®^: 30 mL)	ex vivo	very low	[65]
mycophenolate mofetil (V_D_ (147–364) °; fb (97))	76 (240 min)	0.5, 2.7	CL_AD_: 0.02, 0.07; CL_CD_: 0.0, 0.6	HP/30 (CytoSorb^®^: 30 mL)	ex vivo	very low	[65]
tacrolimus (V_D_ (60.9–343) °; fb (99))	30 (240 min)	0.1, 1.8	CL_AD_: 0, 0.8	HP/30 (CytoSorb^®^: 30 mL)	ex vivo	very low	[65]
	77 (30 min)	-	-	HP/250	in vitro	very low	[34]
Other							
levosimendan (V_D_ (14) °; fb (98))	100 (120 min)	-	-	HP/40 (CytoSorb^®^: 60 mL)	in vitro	very low	[51]

° V_D_ originally given in units of L/kg and recalculated for a typical patient weighing 70 kg. Abbreviations: V_D_, volume of distribution; fb, fraction bound; max. % removal, maximal percentage removal; CL_Cyto_, CytoSorb^®^ clearance; CL_AD_, acute dialysis clearance; CL_CD_, chronic dialysis clearance; EC, extracorporeal; BFR, blood flow rate; Ref, reference; NA, not available. References for V_D_ and fb: cyclosporine A, methylprednisolone, mycophenolate mofetil, tacrolimus [25]; 6-mercaptopurine [64]; levosimendan [66].

**Table 9:** Clinical considerations for selected active substances dosing during CytoSorb^®^ use.

Active Substance	Clinical Consideration	Strenght of Clinical Consideration	Quality of Evidence	Ref.
Sepsis and septic shock				
vancomycin	500 mg additionally in the first 120 min, TDM is advised.	conditional	low/moderate	[19]
meropenem	No need for dose replacement, TDM is advised in infections with organisms with high minimal inhibitory concentration.	conditional	low	[21,22]
linezolid	Possible failure to achieve therapeutic plasma concentrations; TDM is advised.	weak	low	[23]
Intoxications				
-	No clinical considerations can be made.	-	-	-
Cardiac surgery				
apixaban	Feasible and safe for use before and during open heart surgery.	weak	low	[7,56]
ticagrelor	Feasible and safe for use before and during open heart surgery.	weak	very low	[57]
Immunosuppression				
-	No clinical considerations can be made.	-	-	-

## Data Availability

The original contributions presented in this study are included in the article. Further inquiries can be directed to the corresponding author.

## Supplementary Materials

The following supporting information can be downloaded at: https://www.mdpi.com/article/10.3390/antibiotics15040409/s1, Table S1: PRISMA 2020 for Abstracts Checklist; Table S2: PRISMA 2020 Checklist; Table S3: Quality appraisal of eligible studies (observational human studies)—Newcastle—Ottawa quality assessment scale—cohort studies; Table S4: Quality appraisal of eligible studies (case reports)—JBI checklist for case reports; Table S5: Quality appraisal of eligible studies (animal studies)—SYRCLE’s risk of bias tool for animal studies; Table S6: Quality appraisal of eligible studies (in vitro studies)—ToXRTool; Table S7: Ovid MEDLINE(R) ALL <1946 to 23 March 2026>; Table S8: Pubmed title. Reference [97] is cited in the Supplementary Materials.

## Abbreviations

The following abbreviations are used in this manuscript:

ARC	augmented renal clearance
AUC	area under the curve
BFR	blood flow rate
CL_AD_ (L/h)	acute dialysis clearance (litres/hours)
CL_CD_ (L/h)	chronic dialysis clearance (litres/hours)
CLCyto (L/h)	CytoSorb^®^ clearance (litres/hours)
CLCRRT (L/h)	CRRT clearance (litres/hours)
CLendo (L/h)	endogenous clearance (litres/hours)
CPB	cardiopulmonary bypass systems
C_max_	maximal concentration
C_min_	minimal concentration
C_post_	concentrations after CytoSorb^®^ (minimal concentrations)
C_pre_	concentrations before CytoSorb^®^ (maximal concentrations)
CRRT	continuous renal replacement therapy
EC	extracorporeal
ECMO	extracorporeal membrane oxygenation
EXTRIP	extracorporeal treatments in poisoning
fb	fraction bound
fT	fraction unbound
GRADE	Grades of Recommendation, Assessment, Development, and Evaluation
HP	haemoperfusion
IL-6	interleukin 6
max % removal	maximal percentage removal
MDMA	3,4-methylenedioxymethamphetamine
MIC	minimal inhibitory concentration
PICO	Patients, Interventions, Comparison and Outcomes
PK/PD	pharmacokinetics/pharmacodynamics
PRISMA	Preferred Reporting Items for Systematic Reviews and Meta-Analyses
Ref	reference
STAR	The international Safe and Timely Antithrombotic Removal
TDM	therapeutic drug monitoring
TNF-α	tumour necrosis factor-alpha
t_1/2_ cyto (h, min)	CytoSorb^®^ half-life (hours, minutes)
t_1/2_ CRRT (h, min)	CRRT half-life (hours, minutes)
t_1/2_ endo (h, min)	endogenous half-life (hours, minutes)
V_D_	volume of distribution
% removal	percentage removal
