# Supplementary material for: Antibiotics and Other Drugs Removal by the CytoSorb® Haemoadsorber: A Systematic Review of Available Evidence
Source: Antibiotics (Basel). 2026 Apr 17;15(4):409. doi: 10.3390/antibiotics15040409 (PMC13113826; doi:10.3390/antibiotics15040409)
Supplement: Supplementary file 1 [file antibiotics-15-00409-s001.zip › antibiotics-4205812-supplementary.pdf]

Supplementary materials:

Table S1: PRISMA 2020 for Abstracts Checklist

Section and Topic	Item #	Checklist item	Reported (Yes/No)
**TITLE**			
Title	1	Identify the report as a systematic review.	Yes
**BACKGROUND**			
Objectives	2	Provide an explicit statement of the main objective(s) or question(s) the review addresses.	Yes
**METHODS**			
Eligibility criteria	3	Specify the inclusion and exclusion criteria for the review.	Yes
Information sources	4	Specify the information sources (e.g. databases, registers) used to identify studies and the date when each was last searched.	Yes
Risk of bias	5	Specify the methods used to assess risk of bias in the included studies.	Yes
Synthesis of results	6	Specify the methods used to present and synthesise results.	Yes
**RESULTS**			
Included studies	7	Give the total number of included studies and participants and summarise relevant characteristics of studies.	Yes
Synthesis of results	8	Present results for main outcomes, preferably indicating the number of included studies and participants for each. If meta-analysis was done, report the summary estimate and confidence/credible interval. If comparing groups, indicate the direction of the effect (i.e. which group is favoured).	Yes
**DISCUSSION**			
Limitations of evidence	9	Provide a brief summary of the limitations of the evidence included in the review (e.g. study risk of bias, inconsistency and imprecision).	No
Interpretation	10	Provide a general interpretation of the results and important implications.	Yes
**OTHER**			
Funding	11	Specify the primary source of funding for the review.	No
Registration	12	Provide the register name and registration number.	No

From: Page MJ, McKenzie JE, Bossuyt PM, Boutron I, Hoffmann TC, Mulrow CD, et al. The PRISMA 2020 statement: an updated guideline for reporting systematic reviews. BMJ 2021;372:n71. doi: 10.1136/bmj.n71. This work is licensed under CC BY 4.0. To view a copy of this license, visit https://creativecommons.org/licenses/by/4.0/

Table S2: PRISMA 2020 Checklist

Section and Topic	Item #	Checklist item	Location where item is reported
**TITLE**			
Title	1	Identify the report as a systematic review.	Title page
**ABSTRACT**			
Abstract	2	See the PRISMA 2020 for Abstracts checklist.	PRISMA 2020 for Abstract checklist was prepared separately
**INTRODUCTION**			
Rationale	3	Describe the rationale for the review in the context of existing knowledge.	Third to fifth paragraphs of the 'Introduction' section
Objectives	4	Provide an explicit statement of the objective(s) or question(s) the review addresses.	Final paragraph of the 'Introduction' section
**METHODS**			
Eligibility criteria	5	Specify the inclusion and exclusion criteria for the review and how studies were grouped for the syntheses.	'Materials and methods' section, Inclusion and exclusion criteria
Information sources	6	Specify all databases, registers, websites, organisations, reference lists and other sources searched or consulted to identify studies. Specify the date when each source was last searched or consulted.	'Materials and methods' section, Search strategy
Search strategy	7	Present the full search strategies for all databases, registers and websites, including any filters and limits used.	'Materials and methods' section, Search strategy
Selection process	8	Specify the methods used to decide whether a study met the inclusion criteria of the review, including how many reviewers screened each record and each report retrieved, whether they worked independently, and if applicable, details of automation tools used in the process.	'Materials and methods' section, Search strategy and Inclusion and exclusion criteria, Study selection
Data collection process	9	Specify the methods used to collect data from reports, including how many reviewers collected data from each report, whether they worked independently, any processes for obtaining or confirming data from study investigators, and if applicable, details of automation tools used in the process.	'Materials and methods' section, Inclusion and exclusion criteria, Study selection
Data items	10a	List and define all outcomes for which data were sought. Specify whether all results that were compatible with each outcome domain in each study were sought (e.g. for all measures, time points, analyses), and if not, the methods used to decide which results to collect.	'Materials and methods' section, Inclusion and exclusion criteria
	10b	List and define all other variables for which data were sought (e.g. participant and intervention characteristics, funding sources). Describe any assumptions made about any missing or unclear information.	Not reported
Study risk of bias assessment	11	Specify the methods used to assess risk of bias in the included studies, including details of the tool(s) used, how many reviewers assessed each study and whether they worked independently, and if applicable, details of automation tools used in the process.	'Materials and methods' section, Assessment of methodological quality
Effect measures	12	Specify for each outcome the effect measure(s) (e.g. risk ratio, mean difference) used in the synthesis or presentation of results.	Not applicable
Synthesis methods	13a	Describe the processes used to decide which studies were eligible for each synthesis (e.g. tabulating the study intervention characteristics and comparing against the planned groups for each synthesis (item #5)).	'Materials and methods' section, Assessment of methodological quality
	13b	Describe any methods required to prepare the data for presentation or synthesis, such as handling of missing summary statistics, or data conversions.	Not applicable
	13c	Describe any methods used to tabulate or visually display results of individual studies and syntheses.	'Materials and methods' section, Data

Section and Topic	Item #	Checklist item	Location where item is reported
			synthesis
	13d	Describe any methods used to synthesize results and provide a rationale for the choice(s). If meta-analysis was performed, describe the model(s), method(s) to identify the presence and extent of statistical heterogeneity, and software package(s) used.	Not applicable
	13e	Describe any methods used to explore possible causes of heterogeneity among study results (e.g. subgroup analysis, meta-regression).	Not reported
	13f	Describe any sensitivity analyses conducted to assess robustness of the synthesized results.	Not applicable
Reporting bias assessment	14	Describe any methods used to assess risk of bias due to missing results in a synthesis (arising from reporting biases).	Not reported
Certainty assessment	15	Describe any methods used to assess certainty (or confidence) in the body of evidence for an outcome.	Not reported
**RESULTS**			
Study selection	16a	Describe the results of the search and selection process, from the number of records identified in the search to the number of studies included in the review, ideally using a flow diagram.	'Results' section, Study selection
	16b	Cite studies that might appear to meet the inclusion criteria, but which were excluded, and explain why they were excluded.	'Results' section, Figure 1: PRISMA flow diagram
Study characteristics	17	Cite each included study and present its characteristics.	'Results' section, Tables 1 – 4
Risk of bias in studies	18	Present assessments of risk of bias for each included study.	'Supplementary materials'
Results of individual studies	19	For all outcomes, present, for each study: (a) summary statistics for each group (where appropriate) and (b) an effect estimate and its precision (e.g. confidence/credible interval), ideally using structured tables or plots.	Not applicable
Results of syntheses	20a	For each synthesis, briefly summarise the characteristics and risk of bias among contributing studies.	'Results' section, Tables 1 – 4, 'Supplementary materials'
	20b	Present results of all statistical syntheses conducted. If meta-analysis was done, present for each the summary estimate and its precision (e.g. confidence/credible interval) and measures of statistical heterogeneity. If comparing groups, describe the direction of the effect.	Not applicable
	20c	Present results of all investigations of possible causes of heterogeneity among study results.	'Discussion' section, Limitations and strengths
	20d	Present results of all sensitivity analyses conducted to assess the robustness of the synthesized results.	Not applicable
Reporting biases	21	Present assessments of risk of bias due to missing results (arising from reporting biases) for each synthesis assessed.	Not applicable
Certainty of evidence	22	Present assessments of certainty (or confidence) in the body of evidence for each outcome assessed.	'Results' section, Table 5
**DISCUSSION**			
Discussion	23a	Provide a general interpretation of the results in the context of other evidence.	'Discussion' section
	23b	Discuss any limitations of the evidence included in the review.	'Discussion' section, Limitations and strengths
	23c	Discuss any limitations of the review processes used.	'Discussion' section, Limitations and strengths

Section and Topic	Item #	Checklist item	Location where item is reported
	23d	Discuss implications of the results for practice, policy, and future research.	'Discussion' section, Recommendations for future research
**OTHER INFORMATION**			
Registration and protocol	24a	Provide registration information for the review, including register name and registration number, or state that the review was not registered.	Not reported
	24b	Indicate where the review protocol can be accessed, or state that a protocol was not prepared.	Not reported
	24c	Describe and explain any amendments to information provided at registration or in the protocol.	Not reported
Support	25	Describe sources of financial or non-financial support for the review, and the role of the funders or sponsors in the review.	Funding reported at the end of the article
Competing interests	26	Declare any competing interests of review authors.	The authors declare no conflicts of interest
Availability of data, code and other materials	27	Report which of the following are publicly available and where they can be found: template data collection forms; data extracted from included studies; data used for all analyses; analytic code; any other materials used in the review.	Not applicable

From: Page MJ, McKenzie JE, Bossuyt PM, Boutron I, Hoffmann TC, Mulrow CD, et al. The PRISMA 2020 statement: an updated guideline for reporting systematic reviews. BMJ 2021;372:n71. doi: 10.1136/bmj.n71.
This work is licensed under CC BY 4.0. To view a copy of this license, visit https://creativecommons.org/licenses/by/4.0/

Table S3: Quality appraisal of eligible studies (observational human studies) – Newcastle – Ottawa quality assessment scale – cohort studies

Screening questions		Citation				
		Scharf C et al. 2022	Bottari G et al. 2023	Asgarpur G et al.	Roed-Undlien H et al.	Hassan K et al.
Selection	Representativeness of the exposed cohort	1	1	1	1	1
	Selection of the non exposed cohort	1	0	0	0	0
	Ascertainment of exposure	1	1	1	1	1
	Demonstration that outcome of interest was not present at start of study	1	1	1	1	1
Comparability	Comparability of cohorts on the basis of the design or analysis	2	0	0	0	0
Outcome	Assessment of outcome	1	1	1	1	1
	Was follow-up long enough for outcomes to occur	1	1	1	1	1
	Adequacy of follow up cohorts	1	1	1	1	1
Sum		9	6	6	6	6
Reliability		high quality study/low risk of bias	moderate quality study/ moderate risk of bias	moderate quality study/ moderate risk of bias	moderate quality study/ moderate risk of bias	moderate quality study/ moderate risk of bias

Table S4: Quality appraisal of eligible studies (case reports) – JBI checklist for case reports

Screening questions	Citation										
	Dimski et al. 2020	Bottari et al.	Kohler et al. 2021	Zitoune et al.	Paland 2020	Hartjes et al. 2023	Reuchsel et al. 2022	Schryver et al. 2019	Krakowiak et al. 2024	Lang et al. 2020	Buoncore et al. 2022
Were patient's demographic characteristics clearly described?	YES	YES	UNCLEAR	UNCLEAR	NO	YES	UNCLEAR	UNCLEAR	NO	UNCLEAR	UNCLEAR
Was the patient's history clearly described and presented as a timeline?	NO	NO	YES	NO	NO	NO	UNCLEAR	NO	NO	NO	NO
Was the current clinical condition of the patient on presentation clearly described?	UNCLEAR	YES	UNCLEAR	YES	NO	YES	YES	YES	YES	YES	YES
Were diagnostic tests or assessment methods and the results clearly described?	UNCLEAR	YES	YES	UNCLEAR	UNCLEAR	YES	UNCLEAR	UNCLEAR	YES	UNCLEAR	UNCLEAR
Was the interventions or treatment procedures clearly described	YES	YES	YES	YES	YES	YES	UNCLEAR	YES	YES	YES	UNCLEAR
Was the post-intervention clinical condition clearly described?	UNCLEAR	UNCLEAR	UNCLEAR	YES	UNCLEAR	UNCLEAR	UNCLEAR	UNCLEAR	NO	UNCLEAR	YES
Were adverse events (harms) or unanticipated events identified and described?	UNCLEAR	UNCLEAR	UNCLEAR	YES	UNCLEAR	UNCLEAR	UNCLEAR	UNCLEAR	UNCLEAR	YES	YES
Does the case report provide takeaway lessons?	YES	YES	UNCLEAR	NO	UNCLEAR	UNCLEAR	UNCLEAR	NO	UNCLEAR	UNCLEAR	UNCLEAR

Table S5: Quality appraisal of eligible studies (animal studies) – SYRCLE's risk of bias tool for animal studies

Type of bias	Domain	Citation	
		Schneider et al. 2021	Leber et al. 2023
Selection bias	Sequence generation	UNCLEAR	UNCLEAR
	Baseline characteristics	UNCLEAR	UNCLEAR
	Allocation concealment	UNCLEAR	UNCLEAR
Performance bias	Random housing	UNCLEAR	UNCLEAR
	Blinding	UNCLEAR	UNCLEAR
Detection bias	Random outcome assessment	YES	YES
	Blinding	UNCLEAR	UNCLEAR
Attrition bias	Incomplete outcome data	YES	YES
Reporting bias	Selective outcome reporting	YES	YES
Other	Other sources of bias	UNCLEAR	UNCLEAR
Type of bias	Domain	UNCLEAR	UNCLEAR

Table S6: Quality appraisal of eligible studies (*in vitro* studies) – ToXRTool

Screening questions		Citation								
		Reiter et al. 2002	Biever et al. 2021	König et al. 2019	Kortge et al. 2024	Angheloiu et al. 2020	Koertge A et al. 2018	Angheloiu et al. 2017	Harm et al. 2025	Lang et al. 2020
**Criteria Group I: Test substance identification**	Was the test substance identified?	1	1	1	1	1	1	1	1	1
	Is the purity of the substance given?	1	1	1	1	1	1	1	1	1
	Is information on the source/origin of the substance given?	1	1	1	1	1	1	1	1	1
	Is all information on the nature and/or physico-chemical properties of the test item given, which you deem <u>indispensable</u> for judging the data (see explanation for examples)?	1	1	1	1	1	1	1	1	1
**Criteria Group II: Test system characterisation**	Is the test system described?	1	1	1	1	1	1	1	1	1
	Is information given on the source/origin of the test system?	1	1	1	1	1	1	1	1	1
	Are necessary information on test system properties, and on conditions of cultivation and maintenance given?	1	0	0	0	0	0	0	1	0
**Criteria Group III: Study design description**	Is the method of administration given (see explanations for details)?	1	1	1	1	1	1	1	1	1
	Are doses administered or concentrations in application media given?	1	1	1	1	1	1	1	1	1
	Are frequency and duration of exposure as well as time-points of observations explained?	1	1	1	1	1	1	1	1	1
	Were negative controls included (give also point, if not necessary, see explanations)?	0	0	0	0	0	0	0	1	0
	Were positive controls included (give also point, if not necessary, see explanations)?	1	1	1	1	1	1	1	1	1
	Is the number of replicates (or complete repetitions of experiment) given?	1	1	0	1	1	0	0	1	0

**Criteria Group IV: Study results documentation**	Are the study endpoint(s) and their method(s) of determination clearly described?	1	1	1	1	1	1	1	1	1
	Is the description of the study results for all endpoints investigated transparent and complete?	1	1	1	1	1	1	1	1	1
	Are the statistical methods for data analysis given and applied in a transparent manner (give also point, if not necessary/applicable, see explanations)?	0	0	1	1	1	0	1	1	0
**Criteria Group V: Plausibility of study design and data**	Is the study design chosen appropriate for obtaining the substance-specific data aimed at (see explanations for details)?	1	1	1	1	1	1	1	1	1
	Are the quantitative study results reliable (see explanations for arguments)?	1	1	1	1	1	1	1	1	1
Sum		16	15	15	16	16	14	15	18	14
Reliability		not reliable	not reliable	not reliable	not reliable	not reliable	not reliable	not reliable	reliable without restrictions	not reliable

Detailed search strategies for Ovid MEDLINE and Pubmed

Table S7: Ovid MEDLINE(R) ALL <1946 to March 23, 2026>

#	Searches	Results
1	..nlpx "query=cytosorb","desiredResults=10000","minHitsDivisor=7","permitHyponyms=NO","lowestVocabularySearchLevel=none","phrasesBroken=NO","speedWanted=Limit Length","comment=Including Limited Related Terms","elimEnable=NO","constraintMinTerms=2"	600
2	cytosorb.mp. [mp=ti, bt, ab, tx, ot, nm, hw, fx, kf, ox, px, rx, an, ui, ds, on, sy, ux, mx]	593
3	limit 1 to english language	590
4	limit 3 to yr="1860 - 2025"	574

Table S8: Pubmed

Search number,Query,Sort By,Filters,Search Details,Results,Time,Date
2,cytosorb or Cytosorb or CytoSorb,,"English, from 1000/1/1 - 2025/12/31","(""cytosorb""[All Fields] OR ""cytosorbents""[All Fields] OR ""cytosorb""[All Fields] OR ""cytosorbents""[All Fields] OR ""cytosorb""[All Fields] OR ""cytosorbents""[All Fields]) AND ((1000/1/1:2025/12/31[pdat]) AND (english[Filter]))","1,077",17:10:10,2026/03/24
1,cytosorb,,"English, from 1000/1/1 - 2025/12/31","(""cytosorb""[All Fields] OR ""cytosorbents""[All Fields]) AND ((1000/1/1:2025/12/31[pdat]) AND (english[Filter]))","1,077",17:08:59,2026/03/24
